# Abnormal Endocytosis Resulting from Imbalanced Proteomic Doses in Human Aneuploid Embryos

**DOI:** 10.1007/s43032-026-02074-y

**Published:** 2026-03-23

**Authors:** Rubing Duan, Xiaodong Liang, Ying Yang, Jianghua Guo

**Affiliations:** Jiangmen Central Hospital Reproductive Medicine Department, Jiangmen Guangdong, China

**Keywords:** PGT-A, Aneuploidy, Transcriptomics, RNA seq, GO functional enrichment analysis, Pathway analysis, Endocytosis, Intracellular free amino acid detection

## Abstract

**Objective:**

To explore transcriptome differences between diploid and aneuploidy embryos, identify non-invasive screening targets for aneuploidy embryos, and establish a theoretical basis for a pathogenic model.

**Method:**

RNA sequencing compared transcriptomes of diploid and aneuploid blastocysts, with GO and pathway analysis on differentially expressed genes. Fluorescent probes assessed clathrin-dependent and independent endocytosis in both cell types, while fluorescence quantitative PCR validated endocytosis abnormalities in early aneuploidy development. Additionally, free amino acid content was compared to show how aneuploidy genome imbalance affects endocytosis via changes in osmotic pressure.

**Result:**

RNA sequencing revealed 9731 differentially expressed genes between normal diploids and aneuploidys, with 6134 up-regulated and 3597 down-regulated. KEGG analysis indicated these genes are mainly involved in endocytosis-related pathways. Six genes (PSD3, ARAP2, SMAP2, CBLC, AGAP1, SH3GLB1) showed significant differences (*P* < 0.05) in expression between diploid and aneuploidy groups. Molecular probe analysis and Fluorescence quantitative PCR resultsrevealed reduced clathrin-dependent endocytosis and increased clathrin-independent endocytosis in aneuploidy embryos compared to normal diploids(*P* < 0.05). Additionally, aneuploid embryos showed higher relative abundance of 14 free amino acids, particularly methionine.

**Conclusion:**

The study concludes that early transcriptome differences in aneuploid embryos are centered on endocytosis. Normal diploid embryos primarily use clathrin-dependent pathways, whereas aneuploid embryos favor clathrin-independent pathways. The endocytosis abnormalities in aneuploid cells are due to changes in intracellular osmotic pressure. This study provides a potential target for non-invasive detection of aneuploid embryos and lays a theoretical foundation for the establishment of a pathogenicity prediction model for aneuploid embryos.

## Introduction

The term "aneuploid" refers to a condition in which the number of chromosomes within a set deviates from the diploid number, resulting in either an increase or decrease in the total chromosome count within a cell [1]. This chromosomal abnormality poses significant risks during early embryonic development, potentially leading to adverse outcomes such as recurrent implantation failure, miscarriage, and congenital anomalies [2, 3]. Currently, Preimplantation Genetic Testing for Aneuploidy (PGT-A) is employed as a clinical method to detect aneuploidy. However, this invasive procedure may adversely affect the developmental potential of embryos [4]. Consequently, there is an urgent need to develop a safer and more efficient method for screening embryos with normal chromosomal ploidy. Although embryos cultured in vitro secrete trace amounts of metabolites, their DNA content is minimal and challenging to detect. Additionally, the presence of human or animal-derived proteins in the culture medium results in low protein specificity. However, mRNA amplified through transcription meets the dual requirements of high content and specificity, offering a promising avenue for the non-invasive detection of aneuploid conditions.

In contrast to prior research focusing on karyotype-specific aneuploidies [5], such as trisomy 21 and trisomy 18, this study investigates the expression patterns of common aneuploidy gene expression (CAGE) in aneuploid cells. We employed RNA sequencing to compare the transcriptomic profiles of aneuploidy and normal diploid cells, identifying significant differences primarily related to endocytosis through gene ontology analysis. Additionally, we utilized specific molecular probes to characterize the disparities in endocytic processes between diploid and aneuploidy cells. High-performance liquid chromatography (HPLC) was used to quantify free amino acids in aneuploidy cells, revealing that imbalanced aneuploidy genomes result in an increased production of intracellular free amino acids. This imbalance affects conventional clathrin-dependent endocytosis (CDE); however, in contrast to euploid cells, aneuploidy cells achieve compensatory upregulation of the clathrin-independent endocytosis (CIE) pathway by increasing the expression of pleckstrin and Sec7 domain-containing 3 (PSD3). This compensatory mechanism may help maintain normal cellular metabolism and cargo transport.

## Materials and Methods

### Samples

This study was approved by the Reproductive Medicine Ethics Committee of Jiangmen Central Hospital (Ethical Approval No.: Jiangmen Central Hospital Reproductive Ethics Review [2024] No. 034). From January 2024 to August 2025, a total of 2,229 patients underwent assisted reproductive treatment at this center. All embryos used in this study were discarded embryos donated for scientific research after obtaining informed consent from the patients. These cryopreserved discarded embryos were thawed in batches and subjected to trophectoderm biopsy, with the biopsy products undergoing PGT-A testing. Based on the PGT-A results, the donated embryos were classified as euploid, aneuploid, and mosaic embryos. Only euploid and aneuploid embryos proceeded to subsequent research, while mosaic embryos were not included in the scope of this study.

### Controlled Ovarian Hyperstimulation and Embryo Culture

Controlled ovarian hyperstimulation was conducted in accordance with the standard protocols established by our center [6]. Embryos were sequentially cultured in G-1 and G-2 media (Vitrolife, Sweden) at 37 °C with 6% CO2. The embryos utilized in this study were surplus embryos, donated by patients for scientific research purposes, with ethical approval granted under Jiangmen Central Hospital Ethics Review [2024] 034A.

### Embryo Biopsy and Transcriptome Sequencing

#### Embryo Biopsy and Catheterization

After thawing the embryos, laser biopsy was performed, and 5–10 trophoblast cells were taken from each embryo and placed in tubes under a microscope.

#### Nuclear Cytoplasmic Separation

After placing the cells in the sample preservation solution (5 μ L), centrifuge for 30 s and let them stand at 4 ℃ for 5 min. Add 2.5 μ L of magnetic bead premix (2.3 μ L lysis buffer + 0.2 μ L magnetic beads), incubate at room temperature for 1 min after transient dissociation, and place on a magnetic rack for adsorption for 5 min. Take 4 μ L of supernatant as cytoplasm for subsequent RNA seq related experiments. In the remaining liquid, magnetic beads adsorb cell nuclei for subsequent PGT-A related experiments.

#### PGT-A Detection and RNA Seq Sequencing

PGT-A testing is used to distinguish between normal diploid embryos (n = 3) and non diploid embryos (n = 3). In this study, diploid embryos were defined as those with a non diploid rate between 0–20%; Non diploid embryos are defined as those with a non diploid rate ranging from 80 to 100%. Embryos with aneuploidy rates ranging from 20 to 80% are chimeric embryos and were not included in the scope of this study. The RNA Sequencing between diploid and aneuploid were completed by Yikon Genomics Company, Ltd..

### Bioinformatics Analysis

#### Principal Component Analysis

Perform principal component analysis on transcriptome data using the R software package DUBStepR, with the following parameters: min.cells = 0.01 × ncol (input. data).

#### Identify Differentially Expressed Genes

Firstly, batch effect correction of gene expression values was performed using the R software package sva [7] on the transcriptome data of human normal diploid and non diploid embryos collected in this study, as well as on published transcriptome datasets of human normal embryos [8, 9]. The negative values of the expression matrix after batch effect correction were set to 0 [10]. Next, bilateral t-tests were used to identify differentially expressed genes (DEGs), and multiple t-tests corrected for false discovery rate (FDR) were used to more accurately identify DEGs. Genes that satisfy FDR < 0.05 and have an absolute value of log (fold change) ≥ 1 are identified as differentially expressed genes.

#### Gene Ontology Analysis

In order to investigate the functions of DEGs, we used the R software package GOstats [11] to perform Gene Ontology (GO) functional enrichment analysis on DEGs to identify dysregulated biological processes.

#### Pathway Analysis

In order to explore the pathways involved in DEGs, we used Metascapesoftware [12] to perform KEGG pathway enrichment analysis on the pathways involved in DEGs. A p-value < 0.05 was considered as representative GO and KEGG functions, and then visualized using the R software package ggplot2 [13].

### Detection of Endocytosis

According to whether it depends on clathrin, endocytosis can be divided into clathrindependent endocytosis (CDE) and non clathrin dependent endocytosis (CIE). To verify these two endocytic pathways separately, we used two specific fluorescent molecular probes. Red fluorescent labeled Human Dil Low Density Lipoprotein (LDL) was used to detect CDE, and the probe was purchased from Shanghai Yisheng Biotechnology Co., Ltd. (Cat. No: 20614ES76). Fluorescein isothiocyanate labeled folate (FITC-PEG2K-Folic Acid, FA) was used to detect CIE, and the probe was purchased from Xi'an Qiyue Biotechnology Co., Ltd. (Cat. No: R-PF-0822). The working concentration of the above probe is 20 μ g/μ L, incubated at 37 ℃ in the dark for 3.5 h, and then incubated with hocheste33342 staining solution (Cat. No: BL1145A, biosharp) for another 0.5 h. Embryos were rinsed with live cell imaging solution (Cat. No: A596888DJ, Gibco, USA) and examined under a microscope. The microscopy equipment is an Olympus IX73 fluorescence inverted microscope.

### Quantitative Real-Time PCR

#### Sample Collection

The sample sources for fluorescence quantitative PCR are divided into two parts: one part comes from blastocysts that have developed to the fifth to sixth day, and these blastocysts undergo PGT-A detection after biopsy. According to the test results, they were divided into diploid blastocysts (n = 3) and non diploid blastocysts (n = 3), and chimeric blastocysts were excluded from the group. The other part of the fluorescent quantitative PCR samples were obtained from patients' miscarriage products, which were divided into normal group (n = 3) and non diploid group (n = 3, T13, T18, and T22, respectively) based on the results of chromosome karyotyping examination. Chimeric miscarriage products were not included in the group.

#### RNA Extraction

After removing the aborted tissue from the -20 ℃ refrigerator, 300 μ L of RNA extraction solution (Lot No. 2504F048, Servicebio, Wuhan) was added and ground until no large tissue was visible to the naked eye. Then, 1 mL of extraction solution was added and centrifuged at 12,000 rpm at 4 ℃ for 15 min. Take 1 mL of supernatant, add 200 μ L of chloroform and shake vigorously. Let it stand at room temperature for 5 min, centrifuge at 12000 rpm at 4 ℃ for 15 min. Take 350–400 μ L of supernatant and add 500 μ L of isopropanol. Mix well and place in a -20° C refrigerator for 1.5 h. Centrifuge at 12000 rpm at 4° C for 15 min and discard the supernatant. Add 500 μ L of 70% ethanol to the precipitate and wash it. After centrifugation, open the lid to evaporate the alcohol, add 20 μ L of DEPC water to dissolve, measure the concentration, and store at -20 ℃ for later use.

#### cDNAsynthesis

TheRevertAid First Strand cDNA Synthesis Kit was purchased from Guangzhou Fengshuo Biotechnology Co., Ltd. (Lot.No 3183752, Thermo Scientific, USA). The reaction system is shown in Table 1.

**Table 1:** Reverse transcription reaction system

Component	Usage
RT	1μL
RI	1μL
Prime	1μL
dNTP	2μL
Buffer	4μL
DEPC water	9μL
RNA	2μL
Stage1:25℃, 5 min	
Stage2:42℃, 1 h	
Stage3:70℃, 5 min	
Stage4:4℃, for ever	

#### Quantitative Real-Time PCR

The fluorescent quantitative PCR kit was purchased from Shanghai Baisai Biotechnology Co., Ltd. (Lot No…, Takara, Japan). The fluorescence quantitative PCR instrument is Roche LightCycler 96. All primers were synthesized by Shanghai Shenggong Bioengineering Co., Ltd. (Guangzhou Branch). Pre denature at 95 ℃ for 30 s, cycle 40 times at 95 ℃ for 5 s and 60 ℃ for 30 s. At 60 ℃, measure the dissolution curve by increasing the temperature by 0.5 ℃ every 5 s until it reaches 95 ℃. The amplification system is shown in Table 2, and the primer sequences are shown in Table 3.

**Table 2:** Fluorescence quantitative PCR reaction system

Component	Usage
TB GreenPremix Ex Taq	5μL
Forward primer (10 μmol/L)	0.2μL
Reverse primer (10 μmol/L)	0.2μL
DEPC water	3.4μL
cDNA	1μL

**Table 3:** Primer sequence

Gene	Forward	Sequence(5’ to 3’)
*Psd3*	F	AACACGGCTAGAAGCTCATTC
	R	TCCAGCGTCATTCCTGTAAAATC
*Arap2*	F	GTGAAGGACTGTGCAGCAATA
	R	TGGGCTAGATGTCTGAACACTAC
*Smap2*	F	CAGCGCCTGTCATGGATTTG
	R	CTTCTCTAGGGTATTGCTGGTCT
*Cblc*	F	GCGCCTAGAAGAGCAATGC
	R	CTCGTCGTTGGCACTCCTT
*Agap1*	F	CGCTGAGTCGATCTGTCCC
	R	CTCTGTCCATCAACGACAATCTC
*Sh3glb1*	F	ATTACCAGACTTCTGCTAGAGGG
	R	GGATGGAAAACTTCCCAGTTGTT
*Chathrin HC*	F	ATTCTGCCAATTCGTTTTCAGGA
	R	GCTTTCAGTGCAATTACTTTGCT
*Cavealin1*	F	GCGACCCTAAACACCTCAAC
	R	ATGCCGTCAAAACTGTGTGTC
*Gapdh*	F	GGAGCGAGATCCCTCCAAAAT
	R	GGCTGTTGTCATACTTCTCATGG

### Detection of Free Amino Acids in Cells

The levels of free amino acids in cells were detected separately in embryos and aborted tissues, and both samples underwent chromosome ploidy testing and were divided into diploid (normal diploid) and aneuploid groups. The sample size for both diploid and non diploid embryos is 8.The detection was completed by Beijing Qingxiang Research Institute using HPLC method [14].

### Statistical Analysis

Each experiment should be repeated at least three times. Data analysis was conducted using GraphPad Prism 8 software, and statistical results were expressed as x ± SD (unless otherwise specified); Use Student's t-test or one-way ANOVA for significance analysis.

## Results

### Characteristics of the Transcriptome in Normal Human Blastocysts

**Fig. 1:**
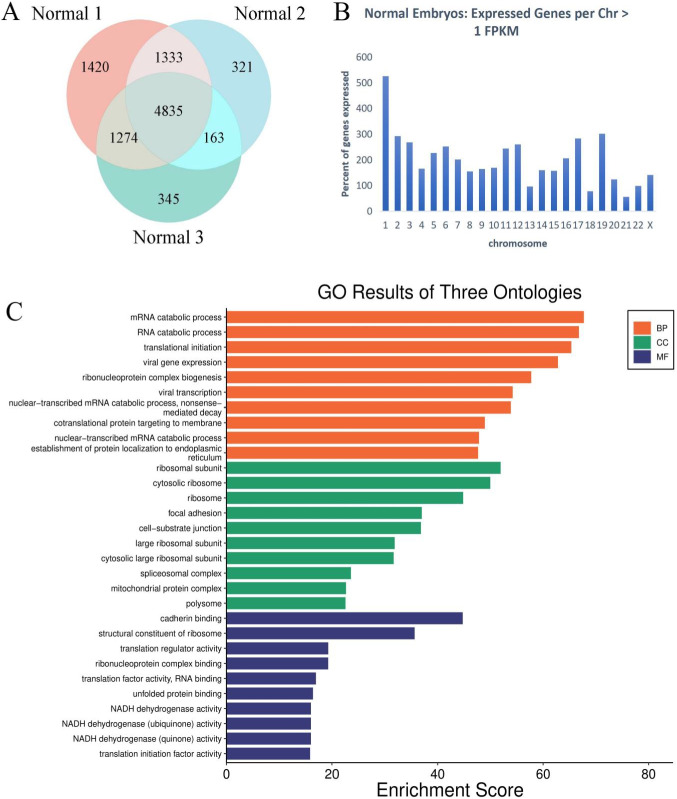
Normal human blastocyst transcriptome characteristics. **A** Venn diagram of commonly expressed protein-coding genes. **B**. The percentage of expressed genes per chromosome. **C**. GO functional enrichment analysis. *BP* Biological process. *CC *Cellular component. *MF *Molecular function

In this experiment, three normal diploid blastocysts were utilized to investigate their transcriptomic characteristics. We identified 4,835 co-expressed transcripts across all embryos, employing a threshold of Fragment Per Kilobase of transcript per Million mapped reads (FPKM) > 1 for gene expression. The overlap of genes expressed in all three embryos was substantial, with 54.56% to 73.07% of genes commonly expressed (Fig. 1A). We calculated the percentage of genes expressed on each chromosome and normalized these values based on the number of genes present on each chromosome (Fig. 1B). The data indicate that there is no significant overexpression or underexpression across the 22 autosomes. Subsequently, we conducted Gene Ontology (GO) functional enrichment analysis on the 4,835 genes expressed in the three normal blastocysts (Fig. 1C). This analysis revealed associations with biological processes such as "translation initiation," "ribonucleoprotein complex biogenesis," "cotranslational protein targeting to membrane," and "establishment of protein localization to the endoplasmic reticulum," which aligns with the active protein synthesis characteristic of this stage of embryonic development.

### Characteristics of the Transcriptome in Aneuploid Embryos

**Fig. 2:**
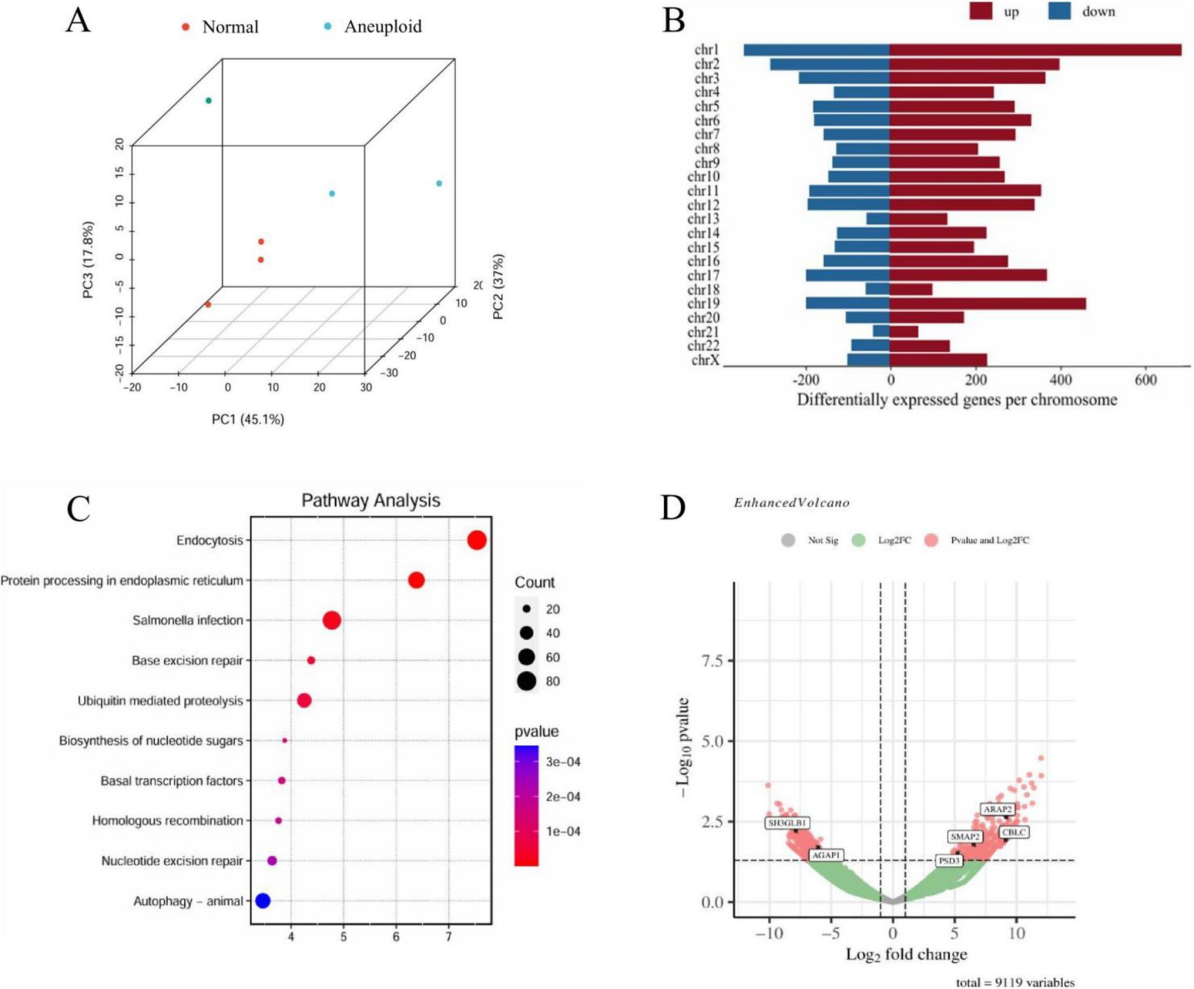
Transcriptomic characteristics of aneuploid blastocysts **A**. Principle component analysis shows separation between aneuploid and normal embryo samples. **B**. Bar plot displaying the number of genes differentially expressed genes per chromosome, with the red and blue coloring indicating up and down regulation respectively. **C**. Pathway analysis (The first term is endocytosis). **D**. Volcano plot displaying the significant genes color coded by red and green, indicating thresholds of adjusted p-value less than 0.05 and greater than log2 fold-change of 2

To examine the transcriptional disparities between diploid and aneuploid embryos, we conducted a comparative analysis of gene expression profiles between normal diploid embryos (n = 3) and aneuploid embryos (n = 3) utilizing RNA sequencing. The three-dimensional principal component analysis (Fig. 2A) revealed that the gene expression patterns of normal blastocysts (represented by red dots) were relatively clustered, whereas aneuploid embryos (represented by blue dots) exhibited distinct separation from one another. This observation suggests that, as early as the fifth to sixth day post-fertilization, aneuploid embryos already exhibit significant differences in gene expression when compared to their diploid counterparts. A total of 9,731 genes were identified, with 6,134 genes showing upregulation and 3,597 genes showing downregulation. The distribution of upregulated and downregulated genes across each chromosome is depicted using red and blue markers, respectively (Fig. 2B). As anticipated, our analysis revealed the most pronounced upregulation of genes on chromosome 1, which correlates with its substantial gene content. Subsequently, we utilized the KEGG database to conduct pathway enrichment analysis on the differentially expressed genes between diploid and aneuploid individuals (Fig. 2C). Notably, the pathway exhibiting the highest concentration of differentially expressed genes was endocytosis, followed by ubiquitin-mediated proteolysis, autophagy, and other pathways. The volcano plot illustrates six endocytosis-related genes with significant expression differences (Fig. 2D), specifically the upregulated genes PSD3, ARAP2, SMAP2, CBLC, and the downregulated genes AGAP1 and SH3GLB1.

### Validation of Differential Gene Expression Using Quantitative Fluorescence

**Fig. 3:**
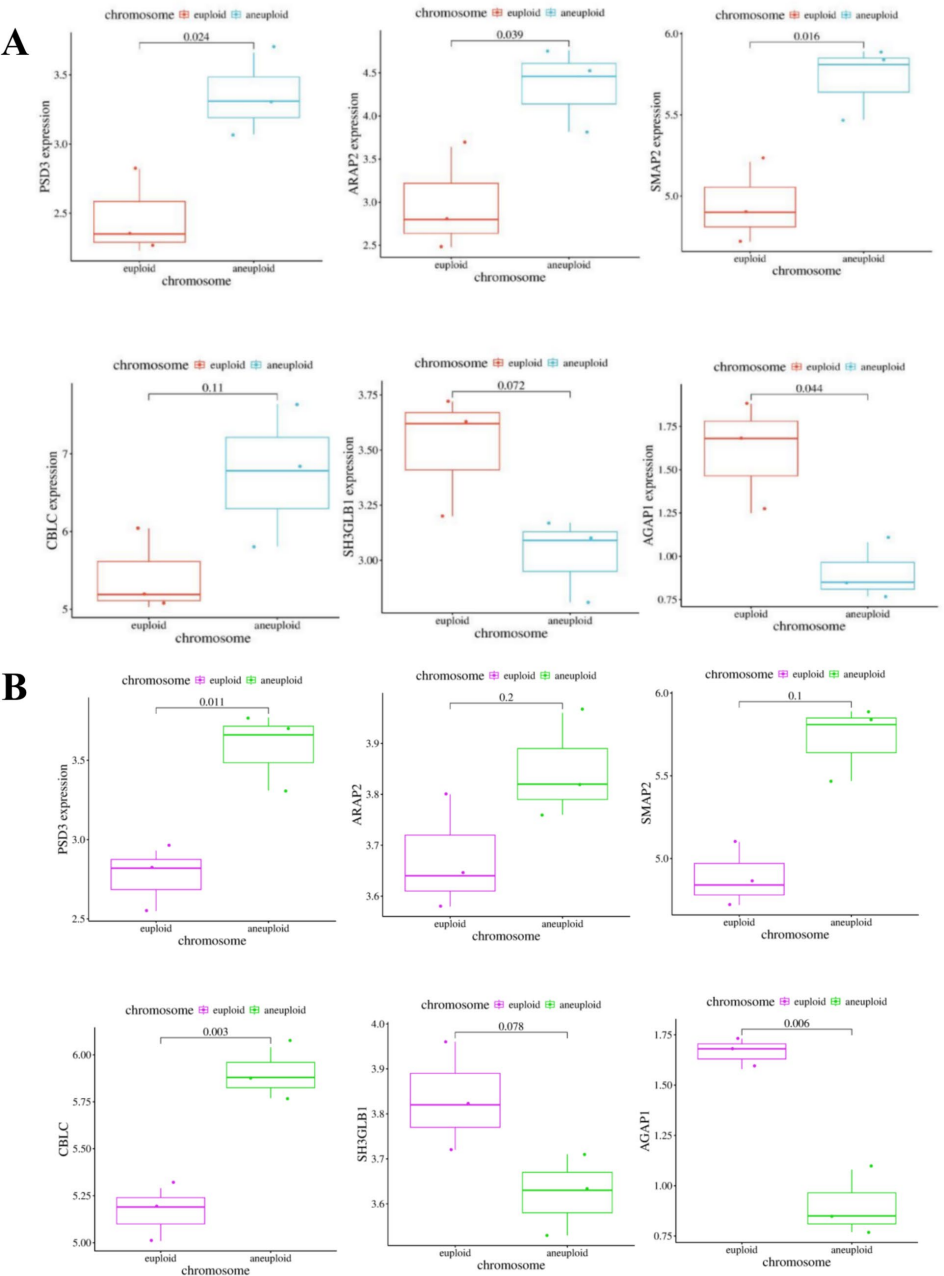
Differentially expressed genes related to endocytosis and their validation **A**. The expression level of differentially expressed genes in miscarriage tissues **B**. The expression level of differentially expressed genes in embryos

Subsequently, we will employ fluorescence quantitative PCR to validate the expression of the six aforementioned genes both in aneuploid miscarriage tissues (Fig. 3A) and embyos (Fig. 3B). Consistent with the findings in embryonic tissue, PSD3, which functions as an activator of Arfs (adenosine diphosphate glycosylation factor, a gene hypothesized to be positively associated with endocytosis), exhibited significantly elevated expression levels in aneuploid compared to normal diploid (*P* < 0.05). Additionally, ARAP2 and SMAP2, both members of the Arfs family, demonstrated a similar upward trend. CBLC and SH3GLB1 are implicated in downstream biological processes of endocytosis, representing ubiquitin-dependent protein degradation and autophagy, respectively.

### Investigation of Endocytosis in Blastocysts and Cleavage-Stage Embryos

**Fig. 4:**
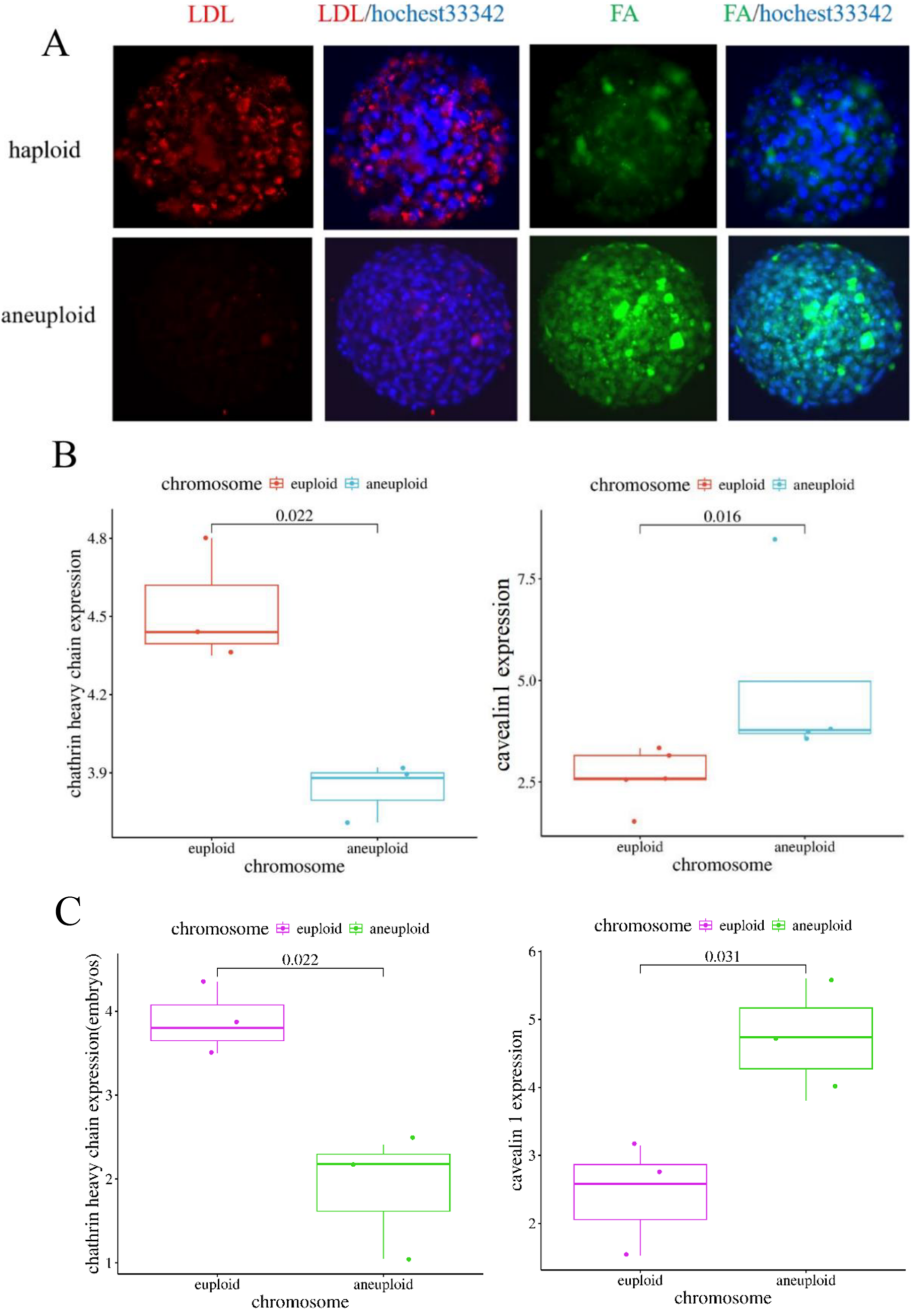
Detection of different endocytic pathways in euploid and aneuploidy **A**. Specific molecular probes detect different pathways of endtosis in euploid and aneuploid. *LDL* Low-density lipoprotein red fluorescent conjugate, indicating clathrin-dependent endocytosis (CDE); *FA* Folic acid-FITC green fluorescent conjugate, indicating clathrin-independent endocytosis (CIE); Hochest33342 Nucleic acid dye, indicating cell nucleus. **B-C**. Fluorescent quantitative PCR detects molecular markers of different endocytic pathways in both miscarriage tissue (**B**) and embyos (**C**)

The aforementioned results indicate that aneuploid embryos may engage in chromosome self-rescue by modulating endocytosis. To test this hypothesis, we employed a red fluorescence-labeled specific probe to examine clathrin-dependent endocytosis (CDE) in both diploid and aneuploid human low-density lipoprotein (LDL), alongside fluorescein isothiocyanate-labeled FITC-PEG2K-Folic Acid (FA) to assess clathrin-independent endocytosis (CIE) (see Fig. 4A). The LDL probe revealed that in normal diploid blastocysts, LDL receptors were predominantly localized on the cell membrane. Following an extended incubation period of four hours, clathrin-dependent endosomes emerged within the cytoplasm. Notably, the quantity of clathrin-dependent endosomes in aneuploid embryos during the same incubation period was significantly reduced compared to that in normal diploid embryos. Conversely, the FA probes demonstrated that normal diploid embryos exhibited lower levels of clathrin-independent endocytosis, whereas aneuploid embryos displayed elevated levels of clathrin-independent endocytosis. The results presented suggest the existence of distinct endocytic pathways between diploid and aneuploidy cells. Specifically, diploid cells predominantly utilize clathrin-dependent endocytosis (CDE), whereas aneuploid cells primarily engage in clathrin-independent endocytosis (CIE). This hypothesis was further substantiated by the detection of CDE and CIE molecular markers in both diploid and aneuploid through fluorescence quantitative PCR (Fig. 4B、C). The clathrin heavy chain gene, which encodes the essential protein clathrin, serves as an indicator of CDE activity. As illustrated in Fig. 4B and 4C, the clathrin heavy chain content in normal diploid is significantly greater than that in aneuploid(*P* < 0.05). Conversely, caveolin, encoded by the caveolin gene and indicative of CIE, is markedly elevated in aneuploid compared to normal diploid (*P* < 0.05). These findings suggest that the differential regulation of endocytic pathways between diploid and aneuploid individuals persists during pregnancy.

### Imbalance in Proteome Dosage in Aneuploid

**Fig. 5:**
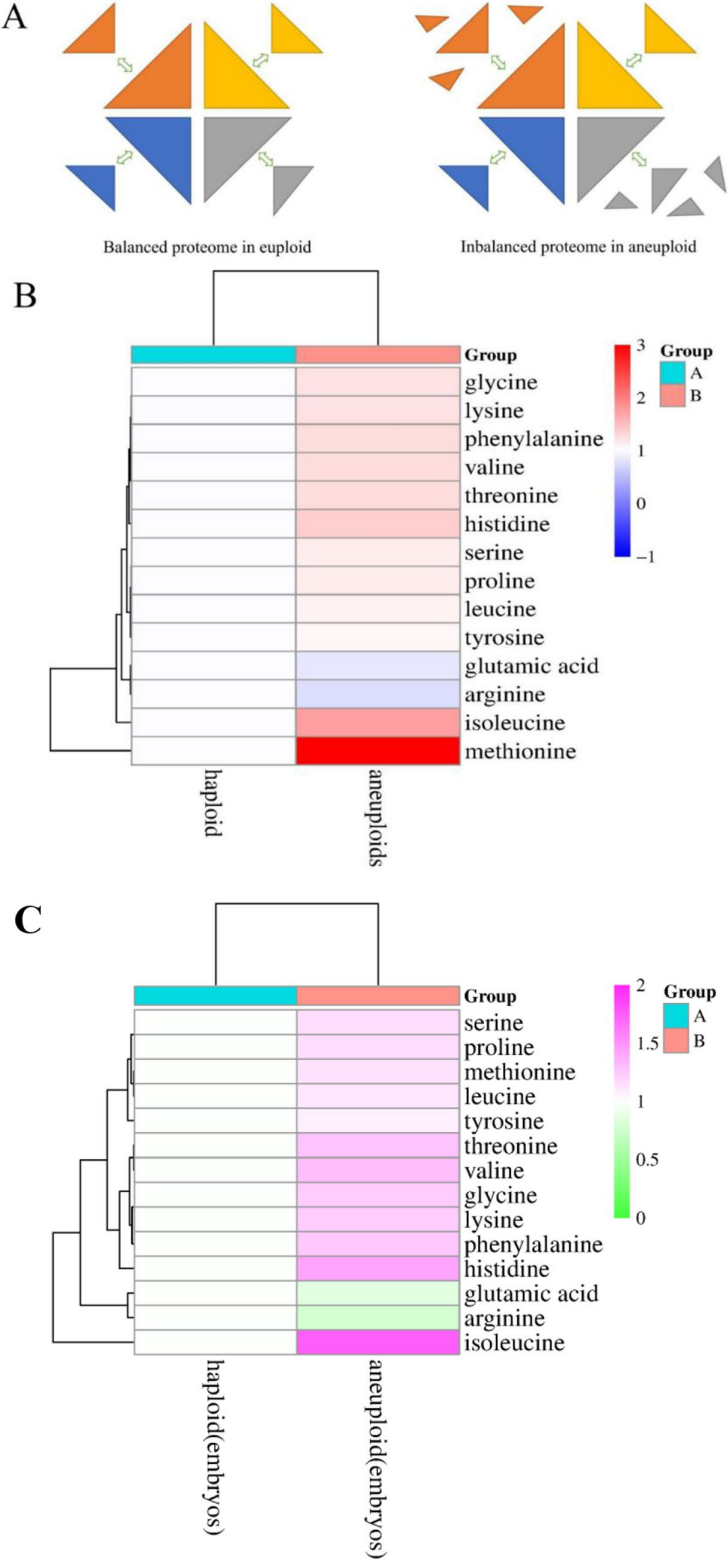
Dose imbalance of the proteome in aneuploidy **A**. Schematic diagram of proteomic dose balance. Left: Prote dose balance in the diploid; right: Proteomic dose imbalance in aneuploid. **B**. Measurement of the intracellular free amino acid abundance in miscarriage tissue. **C**. Measurement of the intracellular free amino acid abundance in embryos

Subsequently, we aim to investigate the impact of genomic imbalance in aneuploidy on endocytosis. In embryos with normal chromosomal ploidy, the chemical stoichiometry of the multi-subunit complexes formed by the encoded proteins is approximately balanced. Following processing and folding, these protein subunits are accurately assembled into functional proteins, as depicted on the left side of Fig. 5A. Conversely, in aneuploid embryos, the imbalance in gene dosage leads to the production of additional proteins, which are unable to form multi-subunit protein complexes due to the absence of complementary subunits, as illustrated on the right side of Fig. 5A. Consequently, free protein subunits accumulate in the cytoplasm, thereby increasing intracellular osmotic pressure. The maintenance of normal cell turgor pressure is essential for endocytosis. To confirm the presence of excessive free amino acids in aneuploid cells, we quantified the levels of 18 free amino acids both in aneuploidy miscarriage tissues (Fig. 5B) and aneuploidy embryos (Fig. 5C). As anticipated, the relative abundance of 14 free amino acids in aneuploid cells was higher than in normal diploid cells, with methionine being particularly elevated. This finding may be attributed to the increased demand for methionine during early embryonic development compared to other amino acids.

## Discussion

Frederick Licciardi and colleagues [15] sought to elucidate the transcriptomic characteristics of haploid, diploid, and polyploid embryos through sequencing, reporting only 3,167 core genes in diploid embryos. In contrast, the present study identified 4,835 core genes in normal diploid embryos, a discrepancy attributed to the limitations of the sequencing technology available at the time, which could only detect genes with an FPKM greater than 10. Upon comparing the transcriptomic differences between diploid and aneuploidy embryos, we observed abnormalities in endocytosis in aneuploidy embryos, findings that align with those of Hung Ji Tsai et al. [16] in their study of aneuploidy yeast and artificially induced aneuploidy leukemia cells. However, Hung Ji Tsai et al.'s research on yeast was confined to general endocytosis. In our study, we further investigated the similarities and differences between clathrin-dependent endocytosis (CDE), primarily reliant on clathrin and represented by endosomes, and clathrin-independent endocytosis (CIE), independent of clathrin and represented by endocytic pits, in diploid and aneuploidy embryos, based on distinct pathways.In our study, we innovatively hypothesized that normal diploid embryos utilize clathrin-dependent endocytosis (CDE) to fulfill their requirements for material metabolism and cargo turnover. In contrast, aneuploidy embryos, due to genomic imbalance, overexpress certain protein subunits that lack corresponding partners, resulting in their accumulation as free amino acids within the cytoplasm (see Fig. 5). This accumulation leads to an increase in intracellular colloid osmotic pressure. The conventional CDE pathway, as described in previous studies [17, 18], is contingent upon normal cellular turgor pressure. However, when cytoplasmic osmotic pressure becomes excessively high, the CDE pathway is impeded, prompting cells to resort to the clathrin-independent endocytosis (CIE) pathway to satisfy their metabolic and cargo transport demands.The aforementioned findings illustrate the adaptive regulatory mechanisms of cells under atypical chromosomal conditions, elucidating the potential molecular foundation for the implantation, sustained gestation, and live birth of certain aneuploidy embryos. Specifically, the upregulation of clathrin-independent endocytosis (CIE) and the downregulation of clathrin-dependent endocytosis (CDE) facilitate the maintenance of essential endocytic processes. Concurrently, the ubiquitin-dependent protein degradation pathway, which operates downstream of endosomes, is adaptively enhanced (see Fig. 2C). This enhancement contributes to the degradation of surplus free amino acids within cells, thereby mitigating the cytoplasmic hyperosmolar state and promoting cellular survival and continued embryonic development. It is imperative that this hypothesis be substantiated through studies with larger sample sizes to ensure its validity.

This study identified six differentially expressed genes: PSD3, ARAP2, SMAP2, and CBLC exhibited elevated expression levels in aneuploidy individuals compared to normal diploid individuals, whereas SH3GLB1 and AGAP1 showed significantly reduced expression in aneuploidy individuals relative to diploid individuals (P < 0.05). PSD3 functions as an activator of Arf6, a pivotal switch molecule involved in endocytosis, and serves as an upstream promoter gene for this process [19]. ARAP2, SMAP2, and AGAP1 are all members of the Arf family, with distribution across various tissues and organs [20].CBLC plays a role in ubiquitination protein hydrolysis [21], and its elevated expression indicates that the imbalance of the aneuploidy proteome may be mitigated through the upregulation of ubiquitination protein hydrolysis, warranting further experimental validation. SH3GLB1 is implicated in autophagy [22], and the occurrence of abnormal autophagy in aneuploidy may represent a self-repair mechanism in embryos; however, additional evidence is required to substantiate this chromosome self-rescue hypothesis. Six differentially expressed genes, namely PSD3, ARAP2, SMAP2, CBLC, SH3GLB1, and AGAP1, present potential targets for the non-invasive detection of aneuploidy and provide a theoretical foundation for the development of a pathogenic prediction model for aneuploidy.

## Conclusion

We have conducted a comprehensive study examining the similarities and differences in transcriptome profiles and amino acid metabolism between normal diploid embryos and aneuploid embryos during early embryonic development. Our findings indicate that the most pronounced difference is observed in the process of endocytosis. Specifically, normal diploid embryos predominantly utilize clathrin-dependent endocytosis pathways for the uptake of signaling molecules and nutrients, whereas aneuploid embryos primarily depend on non-clathrin-dependent endocytosis mechanisms. This regulatory divergence may be associated with the involvement of PSD3, ARAP2, SMAP2, AGAP1, and their downstream effectors, such as CBLC (a ubiquitin-dependent proteolytic enzyme) and SH3GLB1 (an autophagy-related protein). The upregulation of protein hydrolysis may serve to mitigate the increased intracellular osmotic pressure resulting from genomic imbalance, potentially acting as a chromosomal self-rescue mechanism in aneuploidy embryos. This study offers a potential target for the non-invasive detection of aneuploidy embryos and establishes a theoretical foundation for developing predictive models concerning the pathogenicity of chimeric aneuploid embryos.

## Data Availability

Data are available from the authors upon request.

## Declarations

This study has been reviewed and approved by the Reproductive Ethics Committee of Jiangmen Central Hospital (Ethics Approval No.: Jiangxin Medical Ethics Review [2024] 034A). The experimental materials are discarded embryos donated with the patient's written informed consent.
